# Non-Dominant Genetic Algorithm for Multi-Objective Optimization Design of Unmanned Aerial Vehicle Shell Process

**DOI:** 10.3390/polym14142896

**Published:** 2022-07-16

**Authors:** Hanjui Chang, Guangyi Zhang, Yue Sun, Shuzhou Lu

**Affiliations:** 1 Department of Mechanical Engineering, College of Engineering, Shantou University, Shantou 515063, China; 20gyzhang1@stu.edu.cn (G.Z.); suny09222022@163.com (Y.S.); 21szlu@stu.edu.cn (S.L.); 2 Intelligent Manufacturing Key Laboratory of Ministry of Education, Shantou University, Shantou 515063, China

**Keywords:** warpage, volume shrinkage, indentation, uniform sampling method, pareto boundary, genetic algorithm

## Abstract

This paper uses Pareto-optimized frames and injection molding process parameters to optimize the quality of UAV housing parts with multi-objective optimization. Process parameters, such as melt temperature, filling time, pressure, and pressure time, were studied as model variables. The quality of a plastic part is determined by two defect parameters, warpage value and mold index, which require minimal defect parameters. This paper proposes a three-stage optimization system. In the first stage, the main node position of the electronic chip in the module is collected by the unified sampling method, and the chip calculation index of these node positions is analyzed by the mold flow analysis software. In the second stage, the kriging function predicts the mathematical relationship between the mold index and warpage value and the process parameters, such as melt temperature, filling time, packing pressure, and packing time. In the third stage, using LHD sampling and non-dominant rank genetic algorithm II, a convergence curve of warp value is found near the Pareto optimal frontier. In the fourth stage, the fitting degree of Pareto optimal leading edge curve points was verified by analytical experiments. According to experimental verification, it can be seen that the injection molding factors are pressure and pressure time, because the injection molding time and pressure time are completely positively correlated with the mold indicators, the correlation is the strongest, the mold temperature and glue temperature are not the main influencing factors, and the mold temperature shows a certain degree of negative correlation. In this experiment, the die index is mainly improved by injection time and pressure, optimal injection parameter factor combination and minimum injection index, the optimization rate of the die index is up to 96.2% through genetic algorithm optimization nodes and experimental verification, the average optimization rate of the four main optimization nodes is 91.2%, and the error rate with the actual situation is only 8.48%, which is in line with the needs of actual production, and the improvement of the UAV IME membrane is realized.

## 1. Introduction

In-mold electronics (IME) is a new manufacturing technology that combines printing electronics technology with thin film embedded molding technology to produce electronic integrated plastic components. Basically, the input process includes printing, thermos molding, and injection molding. During the thermal forming process, the printed circuit will deform, resulting in the resistance of the circuit. Therefore, the thermal forming process is one of the important processes to determine the performance of IME products.

The injection molding process of the product is completed by two films, in which one film is a decorative film and the other film is a printed circuit, injecting resin between the two films. This new technology, which integrates decorative and electronic functionality, provides designers with a new type of mold decoration method. To put it simply, in-module electronic technology combines printing electronic technology with in-mold decoration technology, turning simple plastic parts decoration products into electronic functional products, and realizing intelligent, self-luminous and touch-type plastic components. The internal structure of such plastic components includes printing sensors, resistance, electronic components, and conductive lines.

**Figure 1:**
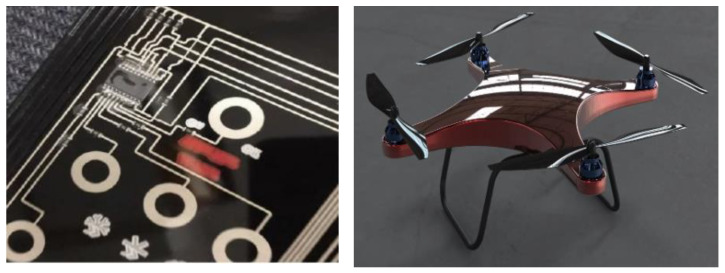
Traditional circuit mode and Drone structure diagram.

The process materials of IME technology include advanced conductive ink, IMD film, sensors, electronic components, injection resin, etc. The typical materials of IME injection resin include high temperature resistance and high-pressure plastic PC, ABS, TPU, etc. Materials composed of IME technology in-mold decoration (IMD) film includes polyethylene terephthalate (PET) film, PC film, PEN film, triacetyl cellulose (TAC) membrane, PI film, etc. Among them, PET film has high surface hardness and good chemical resistance, but the molding performance is poor. PET film will appear with a rebound deformation phenomenon after forming, so it is not suitable to make products with large deformation. Polycarbonate (PC) film has high heat resistance, good tensile performance and processing performance, a large range of molding temperature, but poor fatigue resistance strength, sensitive to the gap, and significant stress cracking resistance. PET film has excellent optical performance, light transmittance of 92%, good colorism, almost no fade and no color under thermal action; the shortcomings are PET products with small surface hardness, easy scratches, large thermal expansion coefficient, high water absorption, and large size expansion caused by temperature and humidity, low gap impact strength, easy to produce stress cracking, as shown in Figure 1.

The purpose of this study was to analyze the relationship between die deviation and warpage of IMD film in the electronic chip foot groove of the injection molding center, so as to obtain the best injection molding conditions, including the melt temperature, filling time, packing pressure, packing time, etc. Process parameters are studied as model variables. The quality of plastic parts is judged by two defect parameters, warpage value and mold index, which need to be minimized. The three-stage optimization system proposed in this paper can effectively solve the problem that the die index is too high in the IMD molding process inside the chip. In the first stage, the unified sampling method was used to determine the influence of the five injection parameters on the defect of the part. In the second phase, a better design solution and better convergence were found near the true Pareto optimal frontier using the non-dominant sorting genetic algorithm II.

## 2. Literature Reviews

In terms of reducing warpage deformation and dent depth and obtaining optimal injection molding parameters, this study needs to involve multi-objective optimization problems, and some population-based global optimization techniques, such as genetic algorithm (GA), Taguchi method, particle swarm optimization algorithm and differential evolution algorithm, have been developed compared with classical mathematical programming. These global optimization techniques have been applied to actual design optimization. Among them, the injection molding parameter data analysis method that is optimized by network prediction and genetic algorithm can obtain the optimal combination of injection molding parameters.

In 2008, in a study by Shia-Chung Chen et al. [1] the grid was printed on the surface of PC films 0.125 mm and 0.2 mm thick, respectively, and the results showed that mold temperature had the most significant effect on the tensile ratio and film thickness change rate. The values for the maximum size and thickness rate of change are not sensitive to film thickness, and the value of a 0.2 mm film is only 0–8% higher than the value of a 0.125 mm film. Multi-step press hardening can reduce residual stress and has a certain effect on reducing the warpage and folding of the forming film. In 2008, C.O. Phillips [2] studied the performance of PC films for IMD processes and films made from PBT and polyethylene terephthalate PET blends during the molding process. The results show that greater molecular flexibility reduces the glass transition temperature of the amorphous phase, increases the possibility of crystallization, and thus reduces the range of the amorphous phase and the decrease in the energy storage modulus of the glass transition. In 2009, Gugyong Kim et al. [3] proposed a computational method to obtain G’Sell parameters using the results of uniaxial tensile tests at constant crosshead velocity to predict the film thickness distribution and graphical changes in the process. The results suggest that by using the rheological parameters and analytical methods developed in this study, the time and cost of trial-and-error methods can be reduced. When using a multi-cavity system, the cavity arrangement and mold configuration can be controlled to obtain an even distribution of film thickness and pattern. In 2010, Shia-Chung Chen et al. [4] developed films in the shape of a mold cavity, and heat transfer on the cavity side was significantly hindered during the molding of IMD parts. In order to study the effect of the film on the temperature field, under the conditions of injection molding of coolant temperature, melt temperature, film material and film thickness, when the thermal conductivity of the film PC increases, the initial film temperature may affect the melt–membrane interface temperature of about 12 °C to 17 °C at the moment of contact between the melt and the membrane, and the melt–membrane interface temperature may change 22.9 °C. The simulated mold temperature field shows reasonable consistency with the experimental results.

In summary, according to previous research, the mechanical behavior and properties of pure PC films in the IMD process and films made of polycarbonate and polyester polybutylene terephthalate (PBT) and PET blends in the molding process were found:

(1). At room temperature, blended membranes have a greater natural tensile ratio and greater elongation before break compared to pure polycarbonate films. This makes them more suitable for cold forming. (2). Among pure polycarbonate films and PC/PBT, the recovery of ambient temperature deformation is most pronounced. PET shows minimal recovery. This suggests that it is most pronounced in parts formed in polycarbonate and least pronounced in parts formed using PET films. During high-speed tensile strain, significant heat is generated in the film; this can lead to thermal softening. Due to the greater molecular flexibility of the polyester component, the polycarbonate blend reacts more quickly to temperature increases. (3). PET films are significantly weaker than PC/PBT films and have greater elongation, so lower molding temperatures can be used. The composition of the polymer and its proportion in the blend determines the glass transition temperature and the associated decrease. Greater molecular flexibility reduces the glass transition temperature of the amorphous phase, but increases the likelihood of crystallization, thereby reducing the range of the amorphous phase, which is why the film made in this article using a PET film is used.

In 2011, A. Martinez et al. [5] studied different textile characteristics used in the pressure drop process to affect the upholstered parts of the fabric. The results from this paper show that using spiral mold technology, the pressure drop is 12–15% higher than that obtained during conventional injection when the plastic flows through the textile, because the polymer must compress the textile foam in addition to being propelled inside the mold. In 2013, D. Lee et al. [6] identified factors that affect the warpage of molded parts for IMD. As a result of the experiment, all molded parts are bent to one side of the decorative film. Melt injection pressure is the main factor affecting the warpage of IMD injection parts. The 908 pieces with angles had the largest amount of warpage, while the 1508 pieces with angles had the smallest warpage. It shows that the warpage of IMD injection molded parts is mainly caused by the unbalanced temperature distribution of the part geometry and the decorative film during the cooling process. In 2013, Hui-Li Che et al. [7] studied the effect of inserted films on asymmetric mold temperature fields during IMD injection molding of polypropylene (PP) parts. The delay was found to cause the mold temperature difference and part warpage to increase as the melt temperature and film thickness increased, and then decreased as the mold temperature increased. By inserting the film, the crystallinity of the molded PP part increases. The asymmetrical cooling system design reduces part warpage when machining with IMD. In 2013, Liam Yueh feng Hsieh et al. [8] proposed a framework that integrates the response surface method and logistic regression 9 to determine the optimal parameter settings for the highest yield of IMD. The RSM is a method of continuously optimizing responses that is widely used in physical experiments. This study conducted an empirical study in collaboration with an IMD company in Taiwan. The results show that the framework can significantly increase the yield of the IMD process from 10% to 87.5%. In 2014, Wei Guo et al. [9] combined finite element flow field analysis, design of experiments, and GA. Based on the central composite design, finite element analysis of different variable combinations was performed, and the mathematical relationship between the dent depth and the variables was established. The incidence of various factors and the interaction between variables on the effect of subsidence markers were studied. Combine the subsidence point prediction model with the genetic algorithm to optimize the variables to minimize the depth of subsidence. In 2014, Jian Zhao et al. [10] proposed a Pareto optimization framework for injection molding process parameters for multi-objective optimization of plastic part quality. The results show that the obtained Pareto fronts are evenly distributed and have good convergence and robustness. Paired Pareto boundaries indicate a significant trade-off between warpage and volumetric contraction, while there is no significant trade-off between dent and volumetric contraction, between dent and warpage.

In 2016, Kun Li et al. [11] proposed a method for optimizing the injection molding process of fiber-reinforced composites by composite backpropagation neural networks and GA. The significance of the influence of each parameter on warpage was studied by the range analysis method. A backpropagation neural network model is established to map the complex nonlinear relationship between design parameters and warpage. Combine genetic algorithms with predictive models to find the optimal process parameter values and significantly improve warpage rate.

In 2019, Wei Guo et al. [12] proposed an IMD injection molding method that combines IMD injection molding with microporous injection molding (MIM). The membrane flattens the uncooled bubbles and turns them to the surface, thereby improving the surface quality of the part. The presence of the film results in an asymmetrical distribution of temperature along the thickness of the specimen, and the higher the temperature on the film side, the cell moves towards it, thereby obtaining a battery offset portion. Specific mechanical properties similar to solids remain unchanged. In 2020, Sang Yoon Lee et al. [13] studied highly ductile conductive inks for three-dimensional in-mold electronics technology, which realizes injection molding and installation of electronic circuits and optical devices through a one-step process, and after the stretchable conductive ink formulation is formed, accurately screen prints circuit patterns and performs a one-step injection molding process. After the high-temperature and high-pressure in-mold electronic process, the conductive filler of the ink circuit is tightly filled, resulting in a significant increase in its conductivity. In 2020, Yao Gong et al. [14] studied the details of how resistance changes with the deformation of printed circuits during thermoforming process. The deformation of the printed circuit is experimentally and numerically characterized, and the regression model between the deformation and the resistance of the circuit is established. Then, according to the numerical simulation results and regression model, the resistance distribution of the circuit after the hot forming process is predicted; during the hot forming process, the printed circuit will be deformed, resulting in a change in the resistance of the circuit, therefore, the hot forming process is one of the important processes that determine the performance of IME products. In 2020, Wei Guo et al. [15] combined MIM with in-mold decoration to produce foam parts with improved surface appearance by varying the heat transfer in the MIM mold. By comparing with the experimental results, it was found that the established model can accurately predict the temperature field. The effect of film on the forming defects of IMD/MIM molded parts was found, and the effect of thin film on the crystallization of IMD/MIM molded parts was revealed. In 2020, Cheng-Hsien Wu et al. [16] studied and designed a series of experimental methods for film molding, thermoforming, and injection molding. The influence of various characteristics on the quality of thermoforming and injection molding processes was determined. For injection molding processes, mold temperature, melt temperature, injection speed and holding pressure are parameters. The injection molding process was optimized using the Taguchi method and the factor that contributed the most was pressure retention. Conductive wires that are decorated in the mold with finished products can be hot-pressed by local heating, and the resistance measured after each process is almost the same. In 2021, Xiang yang Liu et al. [17] developed a new method of screen-printable oxalate ink treatment based on broadband ultraviolet light, which produces traces with excellent electrical properties and is produced in a shorter time compared to thermal sintering. Together, ultraviolet light treatment and silver oxalate molecular inks provide a platform for the rapid production of conductive silver traces on cryogenic substrates and the development of new thermoforming 3D human–machine interface devices, an area of interest to both academia and industry. In 2021, Mehdi Moayyedian et al. [18] used artificial neural networks and Taguchi techniques to solve an optimal set of process parameters. The hierarchical analysis method is used to calculate the weights of each defect of the thin-walled part. The injection molding process of the polypropylene part is simulated, and the optimal set of process parameters proposed is verified. The final product quality is achieved at its best. Filling time has the greatest impact on the quality of the finished product, followed by packing time. The results show that there are uncontrollable parameters in the injection molding process, and the error amplitude of the proposed optimization method is 1.5%. In 2021, Wei Guo et al. [6] used different types of polymer films for IMD/MIM processes. The results showed that the type of membrane had no significant effect on the size of the cells in the transition layer and the mechanical properties of the parts. At a certain film thickness, the core layer offset distance is the largest when using PET film, and the core layer offset distance is the smallest when using film. Similar results were obtained for warpage of parts. The thickness of the transition layer on the membrane side and the bubble marks on the surface of the part change in the opposite way. In 2021, Han-Jui Chang et al. [19] proposed a method for predicting screw process parameters: using crystalline and amorphous polymers as molding materials to predict the injection of composite screws by converting sampling checks that measure delays from real-time and online routine inspections to automated rapid completion methods to create production targets. It can be seen that the recognizable performance evaluation has great advantages in obtaining and analyzing the characteristics of the relevant parameters.

In summary, it can be seen that according to previous studies, the method of GA optimization of injection molding process has an optimization effect that meets the actual requirements. According to the results of previous research, the method of composite GA to optimize the injection molding process of fiber-reinforced composite materials is based on the orthogonal experimental design, and the importance of the influence of each parameter on warpage is studied by the range analysis method. A backpropagation neural network model is established to map the complex nonlinear relationship between design parameters and warpage. Combining genetic algorithms with predictive models to find the optimal process parameter values can significantly improve warpage. The Pareto frontier obtained by the NSGA-II (Non-dominated Soring Genetic Algorithms) algorithm is evenly distributed and has good convergence and robustness. Paired Pareto boundaries indicate a significant trade-off between warpage and volumetric contraction, while there is no significant trade-off between dent and volumetric contraction, or between dent and warpage. A non-dominant genetic algorithm model is established to map the complex nonlinear relationship between design parameters and warpage. Combine genetic algorithms with predictive models to find the optimal process parameter values, reduce the die index and obtain the appropriate warpage values accordingly.

**Figure 2:**
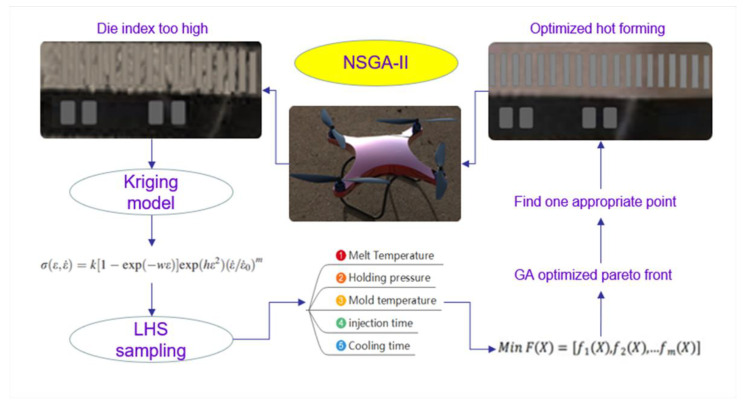
Concept for hot forming experiment.

IME is a new type of manufacturing technology that combines printed electronics with film embedding molding to produce electronically integrated plastic components. Basically, the input method process includes printing, thermoforming, and injection molding. During the press hardening process, the printed circuit will be deformed, resulting in a change in the resistance of the circuit, so the hot forming process is one of the important processes that determine the performance of IME products. Of which, in the main concept shown in Figure 2 and the main contributions of the later chapters of this article are the following five points:

1. The main node position of the electronic chip in the mold is collected by uniform sampling method, and the die index of these node positions is analyzed and determined by the mold flow analysis software, and the die index is improved, in order to reduce the possibility of the IMD film being broken during the product production process due to excessive die index.

2. The kriging function predicts the mathematical relationship between the die index and the warpage value by the process parameters, such as melt temperature, filling time, packing pressure, and packing time as variables and within a certain selection range.

3. The Latin hypercube design (LHD) sampling is then used to find the convergence curve of the warpage value and the die index design near the Pareto optimal frontier by using the non-dominant sorting genetic algorithm II.

4. Verify the fitting degree of Pareto’s optimal leading curve points through analytical experiments.

5. Combined with the results of the front and back diaphragm molding, the molding quality of the product diaphragm is intuitively seen.

## 3. Research Methodology

Previous studies have found that the temperature is distributed asymmetrically along the thickness direction due to the change of the heat transfer coefficient of the melt on the film side. For PC and TPU films, with PET films, the temperature rise at the melt–film interface is faster, and the temperature rise is higher at the end of the melt–film filling stage. The effects of membrane type on the cell structure, forming defects, and mechanical properties of IMD/MIM parts were experimentally studied. The results show that the type of membrane has no significant effect on the size of the cells in the transition layer and the mechanical properties of the part. Under a certain film thickness, the core layer offset distance is the largest when the PET film is used, while the core layer offset distance is the smallest when the TPU film is used. Similar results were obtained for the warpage of the part. The thickness of the transition layer on the film side and the bubble marks on the surface of the part have the opposite changes.

The literature shows that a major difficulty in applying traditional RSM to find optimal parameter settings for IMD/IME is that the output of this process is categorical, the finished product can only be defective or non-defective. In contrast, traditional RSM assumes that the response is quantitative. In order to solve this problem, a non-dominated genetic algorithm technique is proposed in this paper to convert the classification output of IMD/IME into quantitative variables, so as to realize the application of the response surface method.

Compared with the trial-and-error method and the judgment method of domain engineers, the non-dominated genetic algorithm is scientific and systematic, and can quickly identify the optimal parameter setting, saving a lot of time and money. Furthermore, since the proposed framework is an experimental design-based framework, it can handle large-scale problems more efficiently, and the interactions between parameters can also be estimated in the experiments. In this paper, this method is applied to seek the best optimization of injection parameters and at the same time obtain the minimum die index. Key parameters are obtained by identifying characteristic data in the data through the RPE identifiable method.

The reason for choosing the above three materials, mainly in view of the use rate of IMD materials, we selected the most commonly used and stable and reliable three materials for property comparison as shown in Table 1, for the above three common IMD film material characteristics and according to the previous research results, the conclusion was obtainedat room temperature, among PET film, PC films and PBT film, the recovery of ambient temperature deformation is most pronounced. PET shows minimal recovery. This suggests that it is most pronounced in parts formed in polycarbonate and least pronounced in parts formed using PET films. During high-speed tensile strain, significant heat is generated in the film; this can lead to thermal softening. Due to the greater molecular flexibility of the polyester component, the polycarbonate blend reacts more quickly to temperature increases. PET films are significantly weaker than PC/PBT films and have greater elongation, so lower molding temperatures can be used. The composition of the polymer and its proportion in the blend determines the glass transition temperature and the associated decrease. Greater molecular flexibility reduces the glass transition temperature of the amorphous phase, but increases the likelihood of crystallization, thereby reducing the range of the amorphous phase, which is why the film made in this article using a PET film is used.

When designing the depth of the pre-formed mold, the cavity of the pre-formed mold, the pre-formed mold and the injection mold should be matched with each other, and the size and shape of the two should have high machining accuracy. When determining the depth of the pre-formed mold, the extension ability of the membrane itself should be integrated, attention should be paid to avoiding sharp corners at the turning position, and the separation of the membrane and the plastic substrate should be considered. Countermeasures: When designing the product, in order to prevent the height difference of the product from being too large, a step is set to alleviate the change of the drop. At the same time, at the corner of the product, in order to prevent sharp corners, a rounded transition is adopted. Since the circuit board designed this time is a part of the film, PET is used as the material of the film, and the characteristics of the material are:

(1)  E1E2+E2η1ε=(E1+E2)σ+η1σ 

The PET film exhibits viscoelastic plasticity of high polymer. In order to express this characteristic, a three-parameter solid model that can not only reflect the creep characteristics of the high polymer, but also describe the stress relaxation characteristics, is selected. It is composed of a Kelvin model and an A spring is formed in series, and its differential relationship is [2]:

(2) $$\varepsilon .=\frac{d\varepsilon }{\mathrm{dt}}$$

(3) $$v=\frac{\mathrm{dl}}{\mathrm{dt}}$$

where E and B are the elastic moduli of the two springs, and η is the ideal viscosity. According to the Mises yield criterion and the isotropic J, flow theory, the PET material makes the following assumptions when constructing the plasticity criterion of the material. First, assume that the material is isotropic, and select the isotropic hardening model K(q) = σ + Hq to describe the hardening characteristics of the PET film, where σ is the initial yield stress, H is the material hardness, and q is the equivalent plastic strain variable; secondly, when plastic strain occurs, the center of the yield surface is moving, but the yield size of the facets does not change. Finally, the film material is assumed to conform to the isotropic J, flow theory, where m is the stress matrix, γ is the consistency parameter or plasticity multiplier, q is the plastic strain deviator, and K(q) and H(q) are called the isotropic and kinematic hardening moduli, respectively. The general expression for the true strain rate is shown in Equations (2) and (3), where 3 is the extension of the specimen [3]. The nominal strain is shown in Equations (4) and (5), resulting in Equation (6):

(4) e=exp(ε)−1

(5) $$e=\frac{l}{l_{0}}$$

(6) $$\exp\left(\varepsilon \right)=\frac{l+l_{0}}{l_{0}}$$

Differentiation Equation (6) leads to Equation (7).

(7) $$\exp\left(\varepsilon \right)\frac{d\varepsilon }{\mathrm{dt}}=\frac{l}{l_{0}}\frac{\mathrm{dl}}{\mathrm{dt}}$$

Therefore, a relatively simple viscoelastic constitutive law is proposed to express the temperature and rate dependence of the stretching behavior of polymers in the solid state. The advantage of this law is that it can relate the stress response of a viscoelastic material to the strain rate with only four adaptation parameters. The G’Sell constitutive equation for a material subjected to a uniaxial tensile load is given in Equation (8) below:

(8) $$\sigma \left(\varepsilon ,\varepsilon .\right)=k[\left(1-\exp\left(-w\varepsilon \right)\right]\exp\left({h\varepsilon }^{2}\right){\left(\frac{\varepsilon .}{\varepsilon ._{0}}\right)}^{m}$$

According to the above formula, the tensile properties, thickness, bending properties of the film itself and the elongation properties of the circuit itself are combined. When setting its parameters, pay attention to the influence of the shape of the diaphragm, the fillet radius of the die index, the blank holder force, and the gap of die on the drawing depth. In order to avoid the deformation of the product, when designing the thickness of the film, according to the analysis, the thickness should be 0.72 mm, and the wall thickness must be consistent. When solving the stagnation temperature caused by the plastic film, the temperature difference of heat transfer can be calculated through cooling analysis, and the deformation amount can be minimized by compensating the mold temperature in the die side.

Where k is a scaling factor, [1 − exp(−we)] is a viscoelastic term describing the onset of the s–e curve (e¼0 equals 0, rapidly approaching 1 as e increases), and exp(hε) is a large deformation The key reason for the observed strain hardening. The last term expresses the strain rate sensitivity as a power law of exponent m and introduces a reference strain rate e0 for the dimensional uniformity equation uniformity. Equation (1) is based on a constant strain rate, but the experiments performed in this study involved very thin films, which deform when heated, as shown in the peek material properties table (Table 2). In high temperature tensile testing, it is difficult to control the strain rate because it is difficult to measure the change in film quality. Therefore, G’Sell’s model was modified to correspond to a constant crosshead velocity.

**Figure 3:**
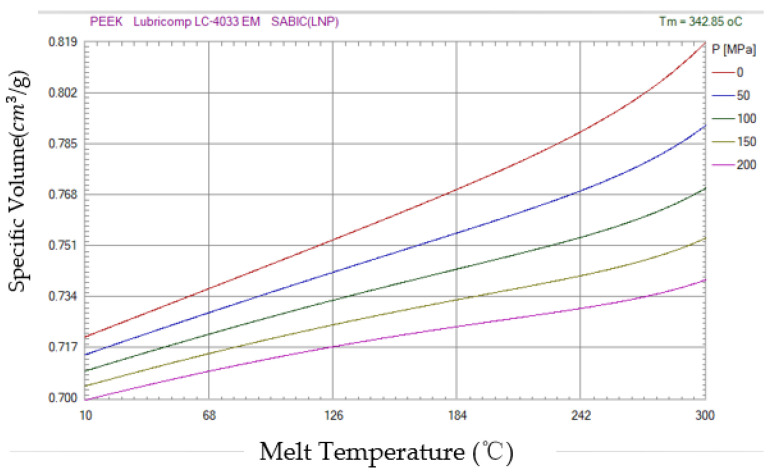
PVT diagram of PEEK of Lubricomp LC-4033EM SABIC material.

The factors affecting the quality of injection plastic film mainly have the following aspects. First, in the injection molding products surface mold decoration process, the plastic film used must have high temperature size stability, transparency, scratch resistance, high temperature resistance, small shrinkage rate and friction resistance and other characteristics. The three-parameter solid model can reflect the creep properties of the polymer and the stress relaxation. According to the three-parameter solid model and J flow theory, a sticky elastic–plastic model of a PET diaphragm and Figure 3 PVT diagram of PEEK material is constructed to characterize the elastic–plastic part of the PET diaphragm in the deformation process. Using thermal transfer technology, the circuit film before molding, place into the mold cavity, then the plastic melts into the mold cavity, make it with the membrane, and the circuit film after leaving the carrier film can permanently stick on the surface of the plastic products, when the melt is curing, opening the mold can gain better decoration; while the carrier film will automatically peel, prepare for the next production cycle. In the design of molding mold depth, mold design, mold and injection molding, mold cavities should cooperate with each other, the size and shape of the two should have high processing accuracy, and in the clear mold depth, they should integrate the extension ability of the film itself; pay attention to avoid sharp angles in the turning position.

**Figure 4:**
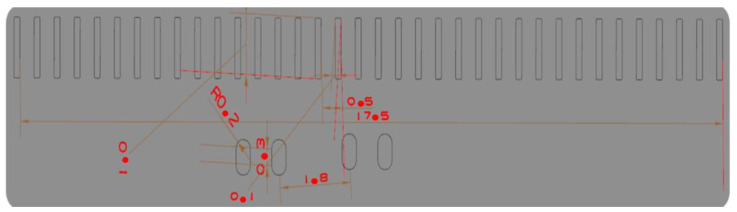
IMD chip important position dimensional drawing.

Combine the tensile performance, thickness, bending performance of the diaphragm itself, and the extension performance of the circuit itself. When setting its parameters, pay attention to the influence of the shape of the diaphragm, the circular angle radius of the convex mode, the pressure edge force, and the gap of the concave and convex modes on the tensile depth. The important position size of the IMD chip is shown in Figure 4, where it can be seen that the internal dimensional accuracy of the chip is very high.

In order to avoid the deformation of the product, the thickness of the design film, according to the analysis, should be 0.72 mm, and the wall thickness must be consistent. When solving the heat stagnation temperature brought by the plastic diaphragm, the temperature difference of the heat transfer can be calculated through cooling analysis, and the deformation of the mold temperature in the concave mold side is compensated. Consider this of the diaphragm and the plastic substrate. When designing products, in order to prevent the drop of the product being large, set up a step to alleviate the change of the drop at the corner of the product. In order to prevent a sharp angle, the round angle transition is adopted. Since the circuit board of this design is part of the film, PET is used as the film material. The characteristics of the material are, namely, a PET diaphragm that shows polymer adhesive elastic plasticity; in order to show this characteristic, a three-parameter solid model that can reflect the creep properties and the stress relaxation properties are selected at 43-5. The three-parameter solid model is composed of a Kelvin model and a spring, whose differential equation is E and B, which are the elastic of the two springs and are the ideal viscosity. According to the yield criterion and isotropic J, flow theory, PET material in the construction of material plasticity criteria, we made the following assumptions: first, assume the material is isotropic, select isotropic hardening model K (q) = + Hq to describe the hardening characteristics of PET film, which is the initial yield stress, H for material hardness, q is the equivalent plastic stress variable.

## 4. Case Study

Combine the tensile properties, thickness, bending properties of the film itself and the elongation properties of the circuit itself. When setting its parameters, pay attention to the influence of the shape of the diaphragm, the fillet radius of the die index, the blank holder force, the gap of the die, etc., on the drawing depth. In order to avoid the deformation of the product, when designing the thickness of the film, according to the analysis, the thickness should be 0.72 mm, and the wall thickness must be consistent. When solving the stagnation temperature caused by the plastic film, the temperature difference of heat transfer can be calculated through cooling analysis, and the deformation amount can be minimized by compensating the mold temperature in the die side.

The selection range of parameters:

As shown in the range of injection molding parameters in Table 3, the factors that affect the quality of injection molding films mainly include the following aspects. First, plastic film. In the in-mold decoration process on the surface of injection molded products, the plastic film used must have the characteristics of high temperature dimensional stability, transparency, scratch resistance, high temperature resistance, low shrinkage, and friction resistance. The three-parameter solid model can not only reflect the creep properties of polymers, but also describe the stress relaxation properties. Therefore, according to the three-parameter solid model and the J-flow theory, a viscoelastic-plastic model of the PET film was constructed to characterize the elastic–plastic part of the PET film in the deformation process.

**Figure 5:**
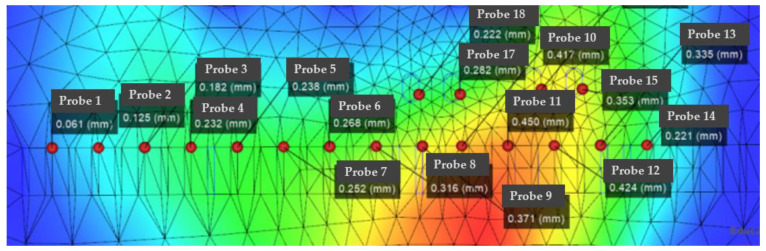
Die index results before optimization.

**Figure 6:**
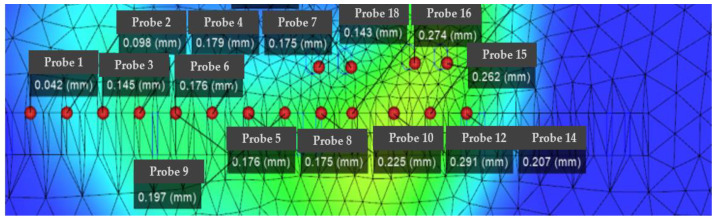
Die index results after optimization.

Using in-mold thermal transfer technology, after forming the pre-made circuit film, put it into the mold cavity, and then inject the plastic melt into the mold cavity to combine with the diaphragm, the circuit film. After leaving the carrier film, it can be permanently bonded to the surface of the plastic product. When the melt is solidified, the mold can be opened to obtain a better decorated product. At the same time, the carrier film will also be automatically peeled off for the next production cycle. In addition, the application of IMD technology can provide help for the consistency of products. The theoretical support for the selection of required materials is as follows:

From the analysis data, it can be concluded that the maximum optimized die index reaches 47%, and the optimized maximum die index is the original target hole size. Secondly, IMD technology will be used to attach a layer of film on the surface of the part. In-mold decoration (IMD) is a kind of efficient, durable, and cost-effective technology; unlike traditional surface printing, IMD sets labels and decorations between film and resin. The printed film is placed on one side of the mold and molten plastic is injected onto the back of the film. The film and plastic are then combined into a single unit, with the trim embedded in it. Compared to other methods, IMD can improve the appearance and durability of the finished product. There are many parameters involved in the manufacturing process of IMDs. If these manufacturing parameters are not set properly, the finished product can easily become defective. Specifically, as shown in Figure 2, the defects of the IMD and the parts that need to be improved are concentrated in the chip socket position. The main method for improvement is to perform factor response surface analysis to obtain the relationship between warpage value, die index and variable factor. In current practice, the parameter settings for IMD are determined based on a trial-and-error approach or the personal experience of domain engineers. Both are biased and can easily lead to low yields. Although the setting of parameters can significantly affect the yield of IMD, its determination is a challenging task. This can be attributed to the following reasons. The analysis of the warpage distribution results is shown in Figure 5. It can be seen that the warpage position is mainly distributed in the center of the part; it is also the part with the smallest thickness of the whole part, and also the part with the largest change in the die index. It is necessary to perform factor analysis and improvement on the defects of this part. IMD analysis results of chip parts before and after optimization and die index before and after IMD mesh analysis and film parameter optimization are shown. The optimized die index is:

IMDs involve many parameters in the manufacturing process. If these manufacturing parameters are not set properly, the finished product can easily become defective. Specifically, as shown in Figure 5, the defects of the IMD and the parts that need to be improved are concentrated in the chip socket position. The main method for improvement is to perform factor response surface analysis to obtain the relationship between warpage value, die index and variable factor. In current practice, the parameter settings for IMD are determined based on a trial-and-error approach or the personal experience of domain engineers. Both are biased and can easily lead to low yields. Although the setting of parameters can significantly affect the yield of IMD, its determination is a challenging task. The analysis of the warpage distribution results is shown in Figure 6. It can be seen that the warpage position is mainly distributed in the center of the part, which is also the part with the smallest thickness of the whole part, and also the part with the largest change in the die index. It is necessary to factor the defects of this part for analysis improvements.

Combine the tensile performance, thickness, bending performance of the diaphragm itself, and the extension performance of the circuit itself. When setting its parameters, pay attention to the influence of the shape of the diaphragm, the circular angle radius of the convex mode, the pressure edge force, and the gap of the concave and convex mode on the tensile depth. Order to avoid the deformation of the product, when designing the thickness of the film, the thickness according to the analysis should be 0.72 mm, and the wall thickness must be consistent. When solving the heat stagnation temperature brought by the plastic diaphragm, the temperature difference of the heat transfer can be calculated through cooling analysis, and the mold temperature in the concave mold side is compensated.

Analysis data can be concluded when the maximum optimization die index reached 47%, optimizing the maximum die index for the original target hole size, then the surface of the parts will use IMD technology attached with a layer of film. In-mold decoration (IMD) is a kind of efficient, durable, and effective technology, unlike traditional surface printing, IMD set labels and decoration between film and resin. The printed film was placed on one side of the mold and the melt plastic was injected into the back of the film. Film and plastic are then combined into a whole and decorated embedded in. IMD can improve the appearance and durability of the finished product when compared to other methods. The IMD is involved in many parameters in the manufacturing process. If these manufacturing parameters are not set properly, the finished product is easily made defective. Specifically, as shown in Figure 3, the defects of IMD and the parts needed for improvement are concentrated in the chip slot position. The main improvement method is to conduct the factor response surface analysis to obtain the relationship between the warping value, the die index, and the variable factors. In current practice, the parameter settings of IMD are determined based on error methods or the personal experience of a domain engineer. Both are biased and can easily lead to low yields. Although the setting of parameters can significantly affect the yield of IMD, its determination is a challenging task. This can be attributed to the following reasons.

The analysis of the warping distribution results is shown in Figure 4. It can be seen that the warping position is mainly distributed in the central position of the part, It is also the part with the smallest thickness of the whole part and the part with the largest change of the die index, which requires the factor analysis and improvement of the defects in this part. This is achieved by IMD grid analysis as well as the die index before film parameter optimization. The IMD is involved in many parameters in the manufacturing process. If these manufacturing parameters are not set properly, the finished product is easily made defective. Specifically, as shown in Figure 2, the defects of IMD and the parts needed for improvement are concentrated in the chip slot position. The main improvement method is to conduct the factor response surface analysis to obtain the relationship between the warping value and the die index and the variable factors. In current practice, the parameter settings of IMD are determined based on error methods or the personal experience of a domain engineer. Both are biased and can easily lead to low yields. Although the setting of parameters can significantly affect the yield of IMD, its determination is a challenging task.

As Table 4 shows, for optimization before and after the key point die index comparison table. the analysis data shown can provide the conclusion that the maximum optimized die index of 47%, the maximum optimized die index is the original target hole size, followed by the use of IMD technology on the surface of the part, will be attached to a film. IMD sets labels and decorations between the film and resin. Place the printed film on one side of the mold and inject the molten plastic onto the back of the film. The film and plastic are then combined into a whole, with decoration embedded in it. Compared to other methods, IMD can improve the appearance and durability of the finished product. IMD involves many parameters in the manufacturing process. If these manufacturing parameters are not set properly, the finished product can easily become a defective product. Specifically, as shown in Figure 1, the flaws in IMD and the parts that need to be improved are concentrated in the chip slot location. The main method of improvement is to analyze the factor response surface to obtain the relationship between the warpage value and the die index and the variable factor. In current practice, IMD parameter settings are determined based on trial-and-error methods or the personal experience of domain engineers. Both are biased and can easily lead to low yields. While the setting of parameters can significantly affect the yield of IMD, its determination is a challenging task. This can be attributed to the following reasons. First, the number of parameters involved in the IMD process is large, resulting in a very large number of possible manufacturing setups. The relationships between IMD parameters are complex, nonlinear, and unknown. To reveal this relationship, only experiments can be conducted. However, experiments are often expensive in terms of time and money. The production output has randomness, that is, under the same parameter settings, this paper analyzes the factor design experiments of the eight parameters of the part injection molding to obtain the result quality response of the in-mold decoration, determines that the parameters can be qualified, and the corresponding combination.

**Figure 7:**
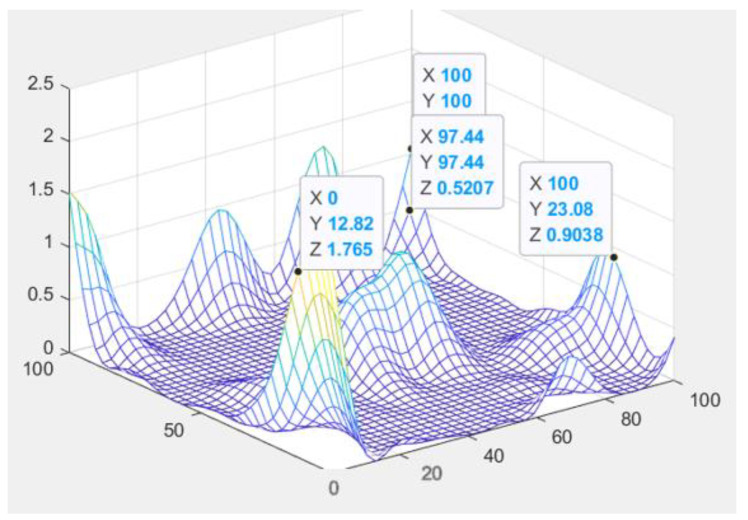
Kriging model predicts surfaces.

After simplifying the multi-objective optimization into single-objective optimization, the kriging model can be used to fit the three-dimensional scatter plot and mean square error plot of the predicted value of each variable for the two defects, as shown in Figure 7. The approximate mathematical relationship between the chip warpage position and the die index can be obtained through the prediction of the response surface.

### LHS Experimental Sampling Point Design

Generally speaking, the higher the number of samples, the more information about the optimization problem, the higher the accuracy of the corresponding approximate model, but the increase in the number of samples will lead to the increase in numerical simulation time, so it is very important to appropriately select the number of design variables according to the specific problem. An LHS experiment is a space-filled experimental design method with high sampling efficiency. The sample combination of design variables is obtained by LHS sampling of design variables, and then numerical simulation using a finite element program to obtain the corresponding response values. This experiment samples four main impact parameters, with a number of 15.

**Figure 8:**
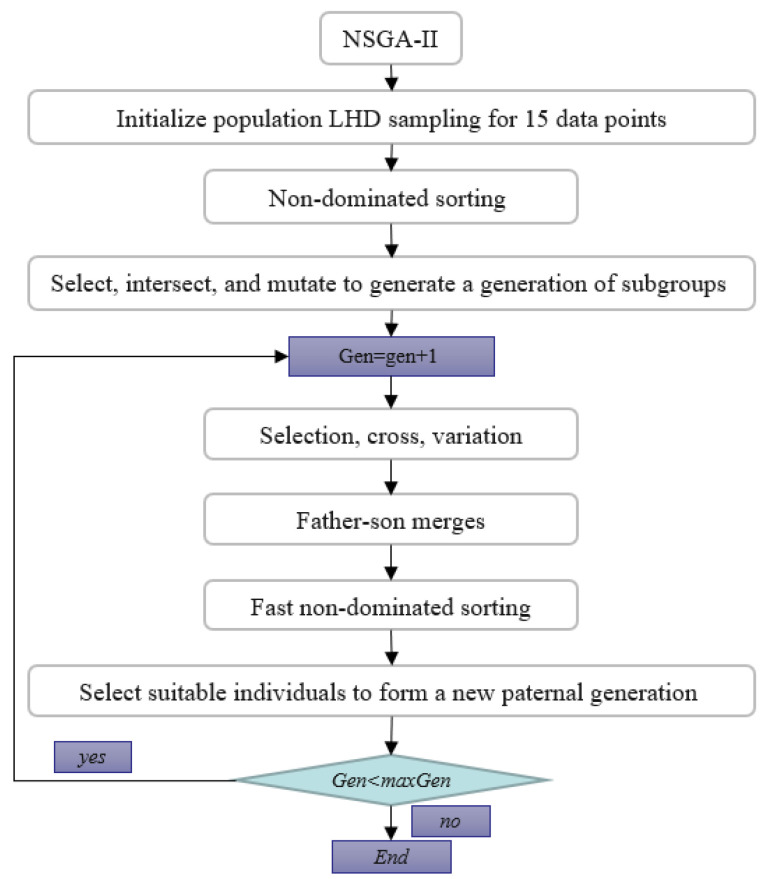
NSGA-II flowchart.

In order to make the sampling as uniform as possible, we introduce some basic concepts of uniform sampling design, considering the test points within the test range. It is also proven that uniform design has better robustness than traditional test design, and uniform design can arrange a test design scheme with more factors, a smaller number of tests, and can reflect the main law of the change of things. Therefore, the uniform test design method mainly includes the following steps: select the test factors, determine the test level, determine the test results of the regression model (using the effectiveness of significance test), analyze the importance of influencing factors, and select sampling points with LHS sampling. Obtain 15 initial sampling points as shown in Table 5. The specific process of the NSGA-II method is shown in Figure 8.

## 5. Demonstration of Kriging Function Model

**Figure 9:**
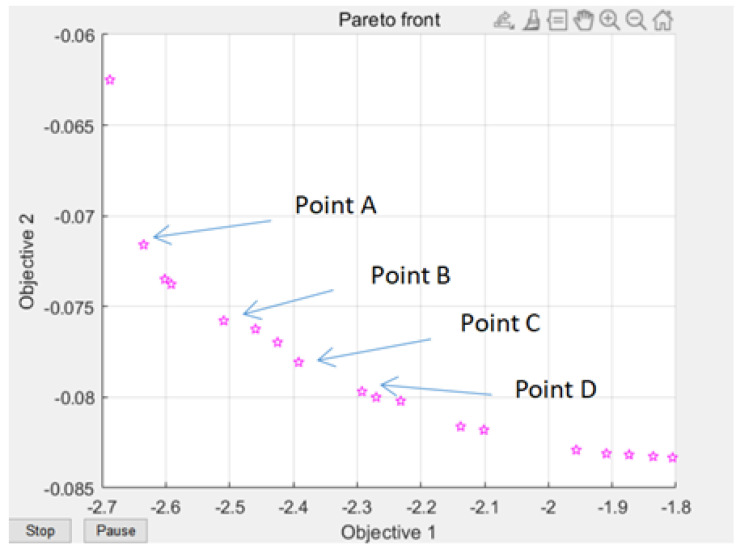
Genetic Algorithm Multi-objective Pareto Iteration Results.

According to the data obtained from the LHS experimental design, the approximate model of the radial basis function can be established. The approximate modeling can be carried out by selecting different kernel function forms, and the accuracy of the approximate model can be determined by the error evaluation. The commonly used indicators are the root mean square error RSME decision, the coefficient R’, and adjustment can determine the coefficient R, and so on. The flow chart is as follows, according to the partial factorial design of four factors, the two-dimensional Pareto boundary and the optimization result of the objective function of die index and warpage value iteratively obtained by genetic algorithm:

We, through the response surface analysis of Figure 9, need warping value and die index and mathematical relationship, and then move into the genetic algorithm multi-objective optimization program analysis experiment. On the genetic algorithm population parameter setting, using NSGA-II method for optimization, floating point coding, set algorithm population size of 100, evolution algebra of 100, 200, 200, 300, cross probability of 0.9, variation probability of 0.1. The optimization results are shown in Figure 9. The Pareto frontiers were obtained at 100, 200, and 300 generations.

From the solution set of the Pareto front, point A above point B is in the relatively sparse position, and the warping is not selected. C\D two points are in the too dense optimal solution range, the results are close to distinguish, and do not chose, the result point below point B tends to be smooth, the warping value in the region of these points is significantly increased, and also do not choose. Therefore, by comparison, the solution of point B is selected as the satisfactory solution, and the warping value and the die index are 2.63 and 0.072, respectively.

## 6. Result and Discussion

**Figure 10:**
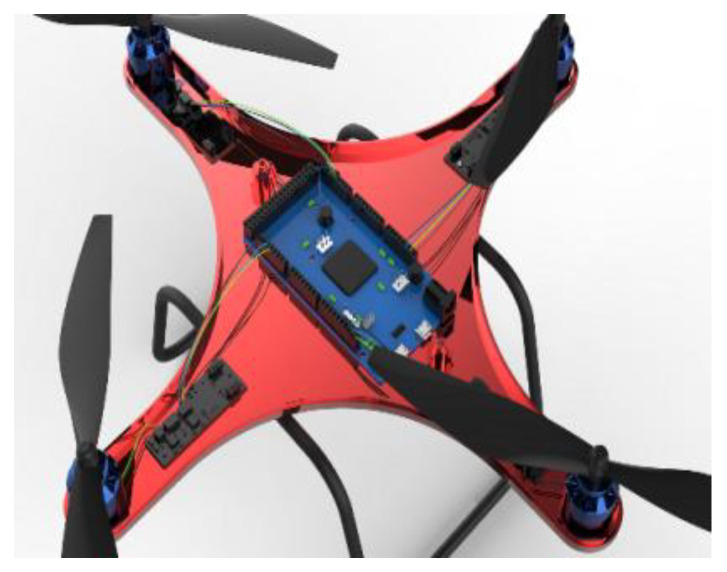
Internal structure of the drone.

This section will discuss the genetic algorithm population multi-objective optimization analysis carried out by the above experiments, and then verify whether the data point values really meet the optimization results through analysis, and obtain our result fitting degree and fitness evaluation. Fitness evaluation is a measure of the quality of the solution, which usually depends on the relationship between the behavior of the solution and the environment. It is usually represented by the objective function. The fitness value of the solution is the main basis for selection in the evolution process. The genetic algorithm basically does not use external information in the process of evolutionary search, and only uses the fitness function as the basis to search by the fitness value of each individual in the population. Therefore, the fitness directly affects the convergence speed of the genetic algorithm and whether it can be found. For the optimal solution, in the selection operation process in the early stage of genetic evolution, some abnormal individuals are usually generated. If the proportional selection method is used, these abnormal individuals control the selection process because of their strong competitiveness, which affects the global optimization performance of the algorithm. In the selection process, that is, when the algorithm is close to convergence, because the individual fitness differences in the population are small, the potential for continued optimization is reduced, and a local optimal solution may be obtained. The above problem we call the genetic algorithm deception problem. The improper design of fitness function may cause this kind of problem, so the design of fitness function is an important aspect of genetic algorithm design. The optimal design of the IME circuit can be clearly seen in the structural physics diagram and the micro circuit structure of the internal structure in Figure 10.

Fitness is a measure of how efficiently an algorithm can reach a solution, this is affected by the behavior of the algorithm and the environment. It is generally expressed as an objective function. The adaptation value of an algorithm is the main basis for selection during the evolution. Genetic algorithms, in general, do not use external information in the evolutionary process but rely on the fitness function. The fitness values of each individual in the population need to be searched, so fitness directly affects the convergence of the genetic algorithm speed and whether the optimal solution can be found. In the beginning of genetic evolution, selection operations often produce some abnormal individuals which, according to the proportional selection method, control the selection process because they are too competitive. Affecting the global optimization performance of the algorithm, in the later selection process of genetic optimization, when the algorithm approaches convergence, due to the small individual fitness difference in the population, the potential to continue optimization is reduced, and a bureau may be obtained for the optimal solution. The above problem is what we call the deception problem of genetic algorithms. The fitness function is not designed properly; this problem may arise, so the design of the fitness function is an important party in the design of genetic algorithms.

**Figure 11:**
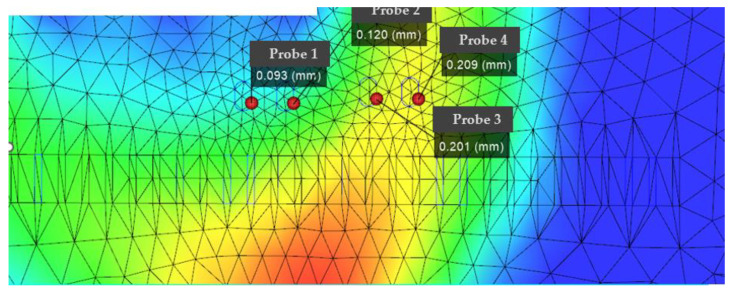
Die flow analysis diagram of the actual die index at point D, in which the smaller value point of the die index in the sampling point: the actual die index at point D: (probe 1).

**Figure 12:**
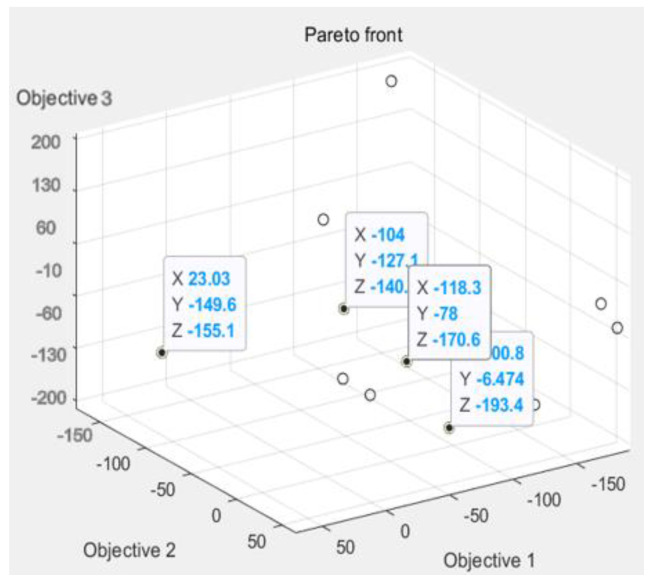
Scatter plot of die index at point D.

**Figure 13:**
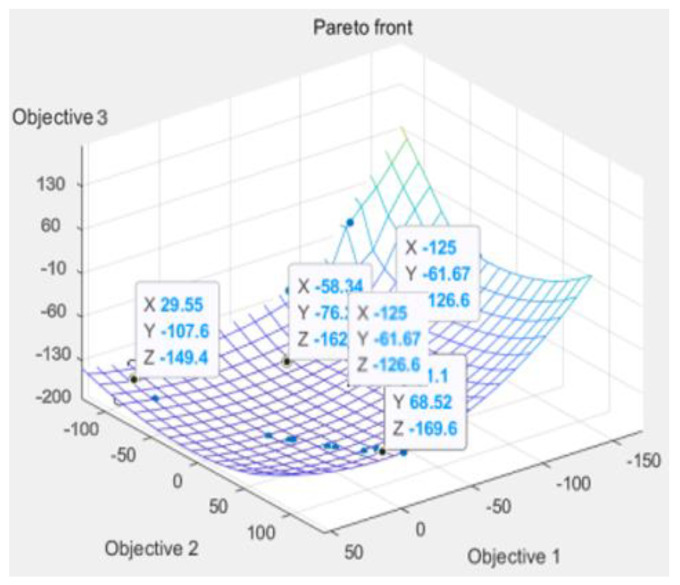
Fitted surface die index at point D.

According to Figure 11, Figure 12 and Figure 13, it can be seen that the distribution of the die index at point D is relatively scattered, and the warpage is in a significantly increased trend range and is in the too dense optimal solution range. The results are similar and difficult to distinguish. The corresponding injection molding parameters are reflected in the injection molding parameter table in Table 6, and the die indexes are 0.201, 0.209, 0.104 and 0.093, respectively. The multi-objective optimization analysis of the genetic algorithm population carried out in the experiment also requires the mold flow analysis to verify whether the data point values really meet the optimization results and obtain our result fitting degree and fitness evaluation. It can be seen that the D point calculated by the algorithm The corresponding injection parameters are sampling point 5:

According to Figure 11, Figure 12 and Figure 13, it can be seen that the distribution scatter position of D point die index is relatively scattered, the warping trend range is in too dense optimal solution range, and the results are close to distinguish. The corresponding injection molding parameters are reflected in the injection molding parameters table (Table 6), and the die index is 0.201, 0.209, 0.104 and 0.093, respectively. The multi-objective optimization analysis of the genetic algorithm population conducted in the above experiments also requires modal flow analysis to verify whether the data point values really meet the optimization results and obtains the fitting degree and fitness evaluation of our results. It can be seen that there is a deviation of 0.19 mm compared with the 0.074 mm calculated by the algorithm, and the adaptation degree is about 75%.

**Figure 14:**
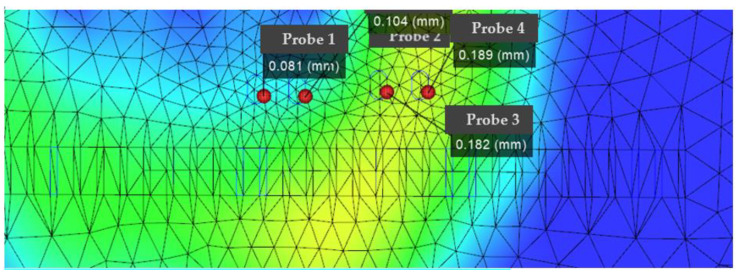
The actual die index flow analysis diagram at point B.

**Figure 15:**
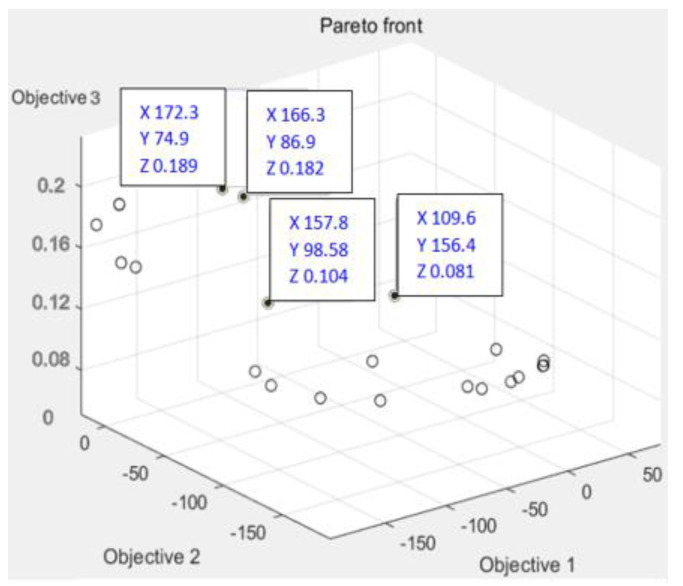
Scatter plot of die index at B.

**Figure 16:**
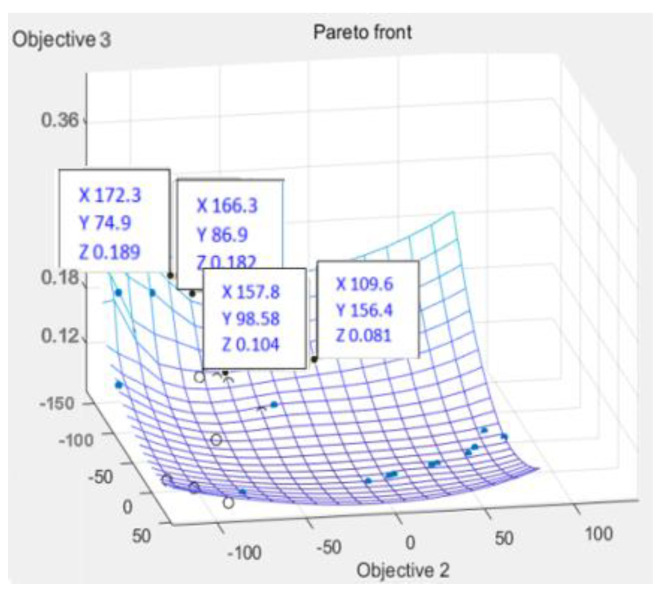
Fitted surface die index at B.

According to Figure 14, Figure 15 and Figure 16, the scattered point of point B die index is concentrated. Compared with the above D points, the die index is in too small optimal solution range, while the D result is similar, and the result point below point B is smooth and the warping value of these points, thus comparing the solution of point B as the satisfactory solution is 0.189, 0.182, 0.104 and 0.081, respectively. The results of the corresponding injection molding parameters are reflected in Table 7.

The multi-objective optimization analysis of the genetic algorithm population conducted in the above experiments also requires modal flow analysis to verify whether the data point value really meets the optimization results and obtains the fitting degree and fitness evaluation of our results. It can be seen that there is a 0.09 mm deviation from the 0.072 mm calculated by the algorithm, and the adaptation degree of this point is about 87.5%.

According to Table 6 and Table 7, the injection molding parameter tables, comparing A, B, C, D, the injection molding factor in Table 8 is the pressure and pressure time, because the injection molding time and pressure time have a completely positive correlation with the die index, the in-mold temperature and glue temperature are not the main influence factors, not is there a completely positive correlation in the result analysis. Therefore, it is concluded that point B is the optimization point of die index, and the die index of this experiment is mainly improved by injection molding time and pressure retaining pressure, and the minimum die index is obtained by the non-dominant genetic algorithm.

**Figure 17:**
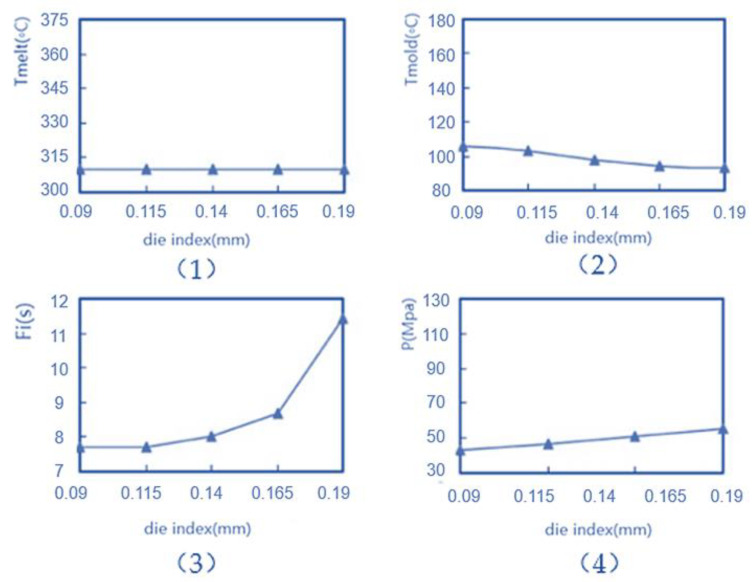
Die exponential relationship line chart.

According to the line chart of the mold index relationship in Figure 17, in Figure 17-(1), as the die index of the mold increases, the melt temperature of (Tmelt) remains basically unchanged in the range of 300–308 °C. In Figure 17-(2), as the die index of the mold increases, the temperature within the mold (Tmold) shows a downward trend, from 109 °C to 84 °C. In Figure 17-(3), as the die index of the mold increases, the filling time (fi) shows an upward trend. In Figure 17-(4), the packing pressure (P) shows an upward trend as the die index of the mold increases. In summary, it can be seen that the injection factors that play a major role in the injection parameters are in-mold temperature (Tmold), filling time (*fi*) and packing pressure (P), and the experimental results show that the melt temperature of (Tmelt) is not strongly correlated with the die index. Among them, the filling time (*fi*) and packing pressure (P) are negatively correlated with the mold stamping index, and the in-mold temperature (Tmold) is positively correlated with the mold stamping index, so it is necessary to appropriately increase the in-mold temperature (Tmold) and reduce the filling time (*fi*) and the packing pressure (P) within a certain range to reduce the mold stamping index value and reduce the possibility of the film being broken in the three main correlation factors, because the filling time (*fi*) and the average slope of the die index image are the largest, so the filling time (*fi*) is the most correlated and requires the largest amplitude of adjustment, while melt temperature is not the main influencing factor and can be fine-tuned. Therefore, the B point is the most advantageous advantage of the mold index, which is mainly improved by adjusting the in-mold temperature (Tmold), filling time (*fi*) and packing pressure (P), and the minimum mold index is obtained by non-dominating genetic algorithms.

According to the experimental results of the die index improvement experiment of Table 9 B and D, it can be concluded that the die index obtained by using the injection molding parameter combination of point B is higher than that of the D point in four nodes, and the highest die index improvement rate is 94.2% and the lowest is 87.5%. The highest die index improvement rate in point B was 88.1% and the lowest was 75%, the average improvement rate of the die index at point B was 91.2%, the improvement rate of point B was 9.4% higher than that of point D, and the injection molding parameters used for point B were melt temperature (Tmelt) 304.62 (°C), in-mold temperature (Tmold) 109.23 (°C), filling time (*fi*) 5 (s) and packing pressure (P) 35.38 (MPa). The injection molding parameters used at point D were melt temperature (Tmelt) 313.59 (°C), in-mold temperature (Tmold) 86.15 (°C), filling time (*fi*) 10 (s) and packing pressure (P) 49.23 (MPa). The comparison that can be obtained is that the filling time (*fi*) and packing pressure (P) and the mold stamping index is negatively correlated, the mold temperature (Tmold) and the mold stamping index is positively correlated, so it is necessary to appropriately increase the in-mold temperature (Tmold), the filling time (fi) and the packing pressure (P) to a certain range, in order to reduce the mold stamping index value, reduce the possibility of film crushing, which is consistent with the above Figure 15 conclusion, and the experimental results meet the requirements.

## 7. Conclusions

**Figure 18:**
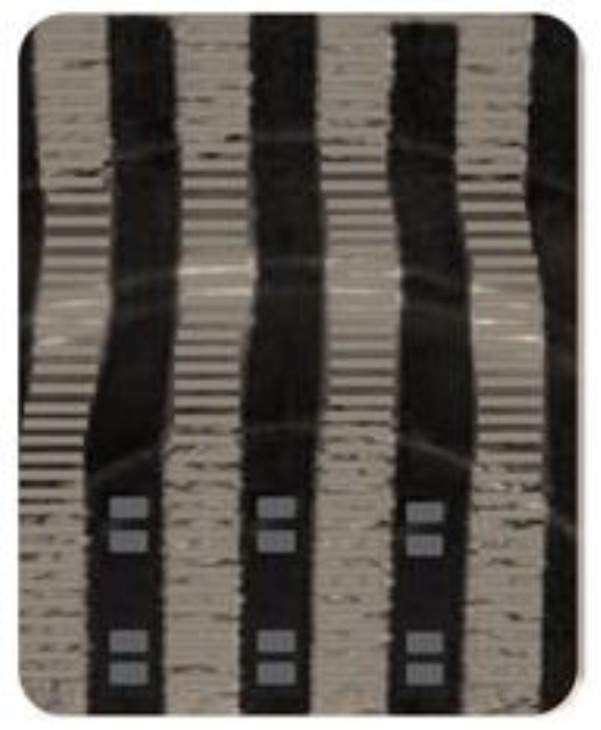
Structure Physical Diagram.

**Figure 19:**
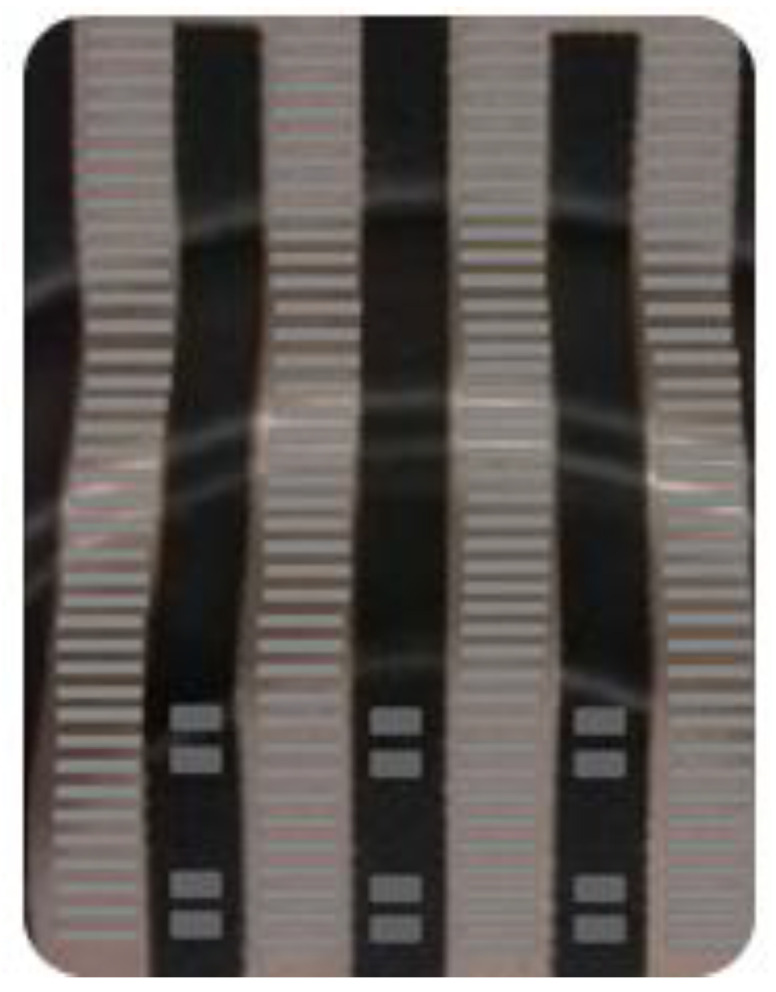
Optimized Structure Physical Diagram.

According to the micrograph of the production experiment in Figure 18, we can see that the die index of the internal diaphragm of the chip before optimization is very large, resulting in a tendency for the diaphragm to crack; Figure 19 is the diaphragm IMD molding effect of the internal circuit of the chip after optimization, which is a great improvement compared with before optimization. There is no trace of diaphragm rupture in the internal circuit pattern, which reflects the optimization effect of chip film blasting of the optimized circuit, which shows that the optimization direction is correct, and the chip index for the actual effect has been greatly improved. Experiments have shown that the mold index of the IMD chip node position inside the drone is mainly improved by reducing the filling time and increasing the filling pressure. The auxiliary index increases the high mold temperature and packing pressure to the appropriate range, and after final optimization, the final product can be obtained without mold bursting, achieving the expected effect.

In the third stage, the sampling point data are analyzed by kriging response surface analysis method, and the warpage value, mold stamping index and mathematical relationship required by the genetic algorithm are obtained, and then the multi-objective optimization program of the genetic algorithm is substituted for analysis experiments.

1. In the overall parameter setting of the genetic algorithm, in the multi-objective optimization based on the NSGA-II method, the floating-point coding method is used to obtain the Pareto boundary.
2. The B-point solution is selected as the satisfactory solution, and the warpage value and the die index are 2.63 and 0.081, respectively. According to the experimental results in Table 10 of the mold die index error rate analysis table, through the results we can verify whether the data point value is really in line with the optimization results and establish our results’ fit and error degree comparison.
3. After multiple production experiments, it can be found that the experimental results at the selected node 1 and the algorithm calculated as 0.072 mm have a deviation of 0.09 mm. The error rate at this point is about 11.1%, and the error rate performance is the best when node 4 is only 3.8%. The resulting data points can be used as the final result of this study.

## Figures and Tables

**Table 1:** Film material property comparison table.

Material	Main Application	Merit	Shortcoming
Polybutylene terephthalate (PBT)	Flat film panel, nameplate	High surface hardness, poor insulation performance and good tensile	High stress, easy to crack, anti-ultraviolet ability
Merlon (PC)	Bulge film panel	High permeability and wear resistance, good toughness good transparency	Corrosion resistance, UV resistance, poor line capacity, high cost
Polyethylene terephthalate (PET)	High quality thin film panels, nameplate	Excellent insulation performance and chemical corrosion resistance	Low transparency, low resistance to UV light, and high cost

**Table 2:** Peek material performance parameter table.

Properties of the Material	
Commercial product name	PEEK
Melt density (g/cm^3^)	1.32
Recommended mold temperature (°C)	155
Recommended melt temperature (°C)	380
Material characteristics	Semi crystalline
Ejection temperature (°C)	85
Modulus of elasticity (MPa)	20,000
Poisson ratio	0.3
Shear modulus (MPa)	90,000

**Table 3:** Injection molding parameter range.

Tmelt (°C)	Fi (s)	P (MPa)	tp (s)	Range
240	2.3	12	1.6	≧
260	2.8	18	3.0	≦

**Table 4:** Die-out index experimental data sheet.

Number of Nodes	Die Index (mm)	Optimized Die Index (mm)	Optimize Efficiency
1	0.061	0.042	31%
2	0.125	0.098	21.6%
3	0.182	0.145	25.5%
4	0.232	0.179	23%
5	0.238	0.175	26.4%
6	0.238	0.176	26.4%
7	0.268	0.175	34.7%
8	0.316	0.175	44.6%
9	0.371	0.197	46.9%
10	0.417	0.225	46%
11	0.450	0.225	50%
12	0.424	0.291	31.3%
13	0.335	0.266	20.6%
14	0.221	0.207	6.3%
15	0.353	0.262	25.7%
16	0.390	0.274	29.7%
17	0.282	0.175	37.9%
18	0.222	0.143	35.6%

**Table 5:** LHS Sample Point Injection Molding Parameter Value Table.

No.	Tmold (°C)	Tmelt (°C)	Fi (s)	P (MPa)
**1**	87.69	316.15	5.64	30.77
**2**	98.46	308.46	9.62	73.85
**3**	83.08	275.13	9.49	60
**4**	120	290.51	9.87	47.69
**5**	90.77	280.26	9.1	38.46
**6**	60	293.08	8.85	27.69
**7**	90.77	280.26	9.1	38.46
**8**	86.15	303.59	10	49.23
**9**	84.62	302.05	8.85	41.54
**10**	109.23	304.62	7.5	35.38
**11**	280.26	9.1	38.46	11.18
**12**	109.23	304.62	5	35.38
**13**	107.77	304.62	6.41	40
**14**	89.23	295.64	8.59	26.15
**15**	96.92	306.46	5.26	69.23

**Table 6:** Point B corresponds to the injection molding parameter table, the actual die index of point D: (probe 1).

Tmelt (°C)	Fi (s)	P (MPa)	Tmold (°C)
313.59	10	49.23	86.15

**Table 7:** Point B corresponds to the injection molding parameter table, and the injection molding parameter corresponding to point B is sampling point 12.

Tmelt (°C)	Fi (s)	P (MPa)	Tmold (°C)
304.62	5	35.38	109.23

**Table 8:** Point A, B, C, D corresponding parameters.

Tmelt (°C)	Fi (s)	P (MPa)	Tmold (°C)	Point	Die Index (max)
300.26	11.4	35.46	84.15	A	0.184 mm
303.59	6.8	49.23	109.23	B	0.098 mm
308.05	8.85	41.54	86.62	C	0.164 mm
304.62	7.5	38.38	90.77	D	0.143 mm

**Table 9:** Analysis for experimental results.

	**Curves Simulate the Die Index**	**B Point Die Index**	**Fit the Index**
probe 1	0.143 mm	0.081 mm	87.5%
probe 2	0.182 mm	0.104 mm	94.2%
probe 3	0.274 mm	0.189 mm	86.8%
probe 4	0.262 mm	0.184 mm	96.2%
Average rate of improvement			91.2%
**Tmelt (°C)**	**Fi (s)**	**P (MPa)**	**Tmold (°C)**
304.62	5	35.38	109.23
	**Curves Simulate the Die Index**	**D Point Die Index**	**Fit the Index**
probe 1	0.143 mm	0.093 mm	75%
probe 2	0.182 mm	0.120 mm	81.7%
probe 3	0.274 mm	0.209 mm	78.5%
probe 4	0.262 mm	0.201 mm	88.1%
Average rate of improvement			80.8%
**Tmelt (°C)**	**Fi (s)**	**P (MPa)**	**Tmold (°C)**
313.59	10	49.23	86.15

**Table 10:** Error analysis of actual production results and experimental results of point B.

	Curves Simulate the Die Index	B Point Impulse Index	Error Rate
probe 1	0.072 mm	0.081 mm	11.1%
probe 2	0.098 mm	0.104 mm	5.8%
probe 3	0.164 mm	0.189 mm	13.2%
probe 4	0.177 mm	0.184 mm	3.8%

## Data Availability

Not applicable.

## References

[1] Chen S.-C., Huang S.-T., Lin M.-C., Chien R.-D. (2008). Study on the thermoforming of PC films used for in-mold decoration. Int. Commun. Heat Mass Transf..

[2] Phillips C.O., Claypole T.C., Gethin D.T. (2008). Mechanical properties of polymer films used in in-mould decoration. J. Mater. Process. Technol..

[3] Kim G., Lee K., Kang S. (2009). Prediction of the film thickness distribution and pattern change during film insert thermoforming. Polym. Eng. Sci..

[4] Chen S.-C., Li H.-M., Huang S.-T., Wang Y.-C. (2010). Effect of decoration film on mold surface temperature during in-mold decoration injection molding process. Int. Commun. Heat Mass Transf..

[5] Martinez A., Castany J., Aisa J. (2011). Characterization of In-Mold Decoration Process and Influence of the Fabric Characteristics in This Process. Mater. Manuf. Process..

[6] Guo W., Yu Z., Wei W., Meng Z., Mao H., Hua L. (2021). Effect of film types on thermal response, cellular structure, forming defects and mechanical properties of combined in-mold decoration and microcellular injection molding parts. J. Mater. Sci. Technol..

[7] Chen H.-L., Chen S.C., Liao W.H., Chien R.D., Lin Y.T. (2013). Effects of insert film on asymmetric mold temperature and associated part warpage during in-mold decoration injection molding of PP parts. Int. Commun. Heat Mass Transf..

[8] Hsieh L.Y., Chang K.-H. (2013). Yield improvement on in-mold decoration manufacturing through parameter optimization. Int. J. Precis. Eng. Manuf..

[9] Guo W., Hua L., Mao H. (2014). Minimization of sink mark depth in injection-molded thermoplastic through design of experiments and genetic algorithm. Int. J. Adv. Manuf. Technol..

[10] Zhao J., Cheng G., Ruan S., Li Z. (2015). Multi-objective optimization design of injection molding process parameters based on the improved efficient global optimization algorithm and non-dominated sorting-based genetic algorithm. Int. J. Adv. Manuf. Technol..

[11] Li K., Yan S., Pan W., Zhao G. (2016). Warpage optimization of fiber-reinforced composite injection molding by combining back propagation neural network and genetic algorithm. Int. J. Adv. Manuf. Technol..

[12] Guo W., Yang Q., Mao H., Meng Z., Hua L., He B. (2019). A Combined In-Mold Decoration and Microcellular Injection Molding Method for Preparing Foamed Products with Improved Surface Appearance. Polymers.

[13] Lee S.Y., Jang H.S., Lee H.K., Kim J.S., Lee S.K., Song H.J., Jung J.W., Yoo E.S., Choi J. (2020). The development and investigation of highly stretchable conductive inks for 3-dimensional printed in mold electronics. Org. Electron..

[14] Gong Y., Cha K.J., Park J.M. (2020). Deformation characteristics and resistance distribution in thermoforming of printed electrical circuits for in-mold electronics application. Int. J. Adv. Manuf. Technol..

[15] Yan K., Gou W., Mao H., Yang Q., Meng Z. (2020). Investigation on Foamed PP/Nano-CaCO3 Composites in a Combined in-Mold Decoration and Microcellular Injection Molding Process. Polymers.

[16] Wu C.H., Li J.H. (2020). The use of 3D in-mold decoration technology to form a film with printed circuits. Polym. Eng. Sci..

[17] Liu X., Li D., Fukutani H., Trudeau P., Khoun L., Mozenson O., Sampson K.L., Gallerneault M., Paquet C., Lacelle T. (2021). UV-Sinterable Silver Oxalate-Based Molecular Inks and Their Application for In-Mold Electronics. Adv. Electron. Mater..

[18] Moayyedian M., Dinc A., Mamedov A. (2021). Optimization of Injection-Molding Process for Thin-Walled Polypropylene Part Using Artificial Neural Network and Taguchi Techniques. Polymers.

[19] Chang H.-J., Zhang G.-Y., Su Z.-M., Mao Z.-F. (2021). Process Prediction for Compound Screws by Using Virtual Measurement and Recognizable Performance Evaluation. Appl. Sci..

